# Highly Sensitive and Specific SARS-CoV-2 Serological Assay Using a Magnetic Modulation Biosensing System

**DOI:** 10.3390/bios12010007

**Published:** 2021-12-23

**Authors:** Shira Avivi-Mintz, Yaniv Lustig, Victoria Indenbaum, Eli Schwartz, Amos Danielli

**Affiliations:** 1 Faculty of Engineering, The Institute of Nanotechnology and Advanced Materials, Bar-Ilan University, Max and Anna Webb Street, Ramat Gan 5290002, Israel; Shiravivi@gmail.com; 2 Central Virology Laboratory, Israel Ministry of Health, Chaim Sheba Medical Center, Tel-HaShomer, Ramat Gan 5262000, Israel; yaniv.lustig@sheba.health.gov.il (Y.L.); Viki.Indenbaum@sheba.health.gov.il (V.I.); 3 Sackler Faculty of Medicine, Tel Aviv University, Tel Aviv 6997801, Israel; elischwa@tauex.tau.ac.il; 4 The Center for Geographic Medicine, Chaim Sheba Medical Center, Tel-Hashomer, Ramat Gan 5262000, Israel

**Keywords:** SARS-CoV-2, COVID-19, magnetic modulation, optical biosensing, serology, fluorescence-based assay

## Abstract

Sensitive serological assays are needed to provide valuable information about acute and past viral infections. For example, detection of anti-severe acute respiratory syndrome coronavirus 2 (SARS-CoV-2) IgG antibodies could serve as the basis for an “immunity passport” that would enable individuals to travel internationally. Here, utilizing a novel Magnetic Modulation Biosensing (MMB) system and the receptor-binding domain of the SARS-CoV-2 spike protein, we demonstrate a highly sensitive and specific anti-SARS-CoV-2 IgG serological assay. Using anti-SARS-CoV-2 IgG antibodies, RT-qPCR SARS-CoV-2-positive and healthy patients’ samples, and vaccinees’ samples, we compare the MMB-based SARS-CoV-2 IgG assay’s analytical and clinical sensitivities to those of the enzyme-linked immunosorbent assay (ELISA). Compared with ELISA, the MMB-based assay has an ~6-fold lower limit of detection (129 ng/L vs. 817 ng/L), and it detects an increase in the IgG concentration much earlier after vaccination. Using 85 RT-qPCR SARS-CoV-2-positive samples and 79 -negative samples, the MMB-based assay demonstrated similar clinical specificity (98% vs. 99%) and sensitivity (93% vs. 92%) to the ELISA test, but with a much faster turnaround time (45 min vs. 245 min). The high analytical and clinical sensitivity, short turnaround time, and simplicity of the MMB-based assay makes it a preferred method for antibody detection.

## 1. Introduction

The gold standard for the diagnosis of coronavirus disease 2019 (COVID-19) uses the reverse-transcription quantitative polymerase chain reaction (RT-qPCR) to directly detect the virus’ ribonucleic acid (RNA) in nasopharyngeal swab samples [1,2]. While RT-qPCR is accurate, its diagnostic efficacy is limited to a few weeks after the infection. Serological assays to detect antiviral antibodies against SARS-CoV-2, namely immunoglobulin M (IgM), immunoglobulin G (IgG), and immunoglobulin A (IgA), are not limited to the first weeks of the disease [3], and they indicate previous or recent SARS-CoV-2 infection, irrespective of whether the individual had a severe, mild, or even asymptomatic infection. IgA and IgM antibodies typically rise several days after SARS-CoV-2 infection, remain high for ~7–14 days, and indicate an acute viral infection. IgG antibodies are usually detected ~14 days after infection, remain detectable for several months, and indicate past viral infection [4,5,6]. Serological tests provide a valuable understanding of the antibody response, seroconversion, infection rate, and virus transmission within populations, and they are essential for assessing and understanding the COVID-19 outbreak.

Recently, it was suggested that the detection of anti-SARS-CoV-2 IgG antibodies could serve as the basis for an “immunity passport” that would enable individuals to travel or to return to work [7]. However, the IgG levels and kinetics among SARS-CoV-2-positive individuals vary significantly, mainly between symptomatic and asymptomatic people [4,5]. For example, compared with symptomatic SARS-CoV-2-positive individuals, asymptomatic individuals exhibit lower levels of IgG antibodies [8]. Moreover, in asymptomatic individuals, anti-SARS-CoV-2 IgG antibodies decline faster over time: in many cases, after three to four months, the result is a complete seroreversion [8]. According to the Centers for Disease Control and Prevention (CDC), up to 80% of SARS-CoV-2-positive individuals are asymptomatic [9], and a possible decrease in the level of their SARS-CoV-2 antibodies may increase reinfection. Hence, to characterize SARS-CoV-2 infection immunogenicity and allow governments to issue “immunity passports”, periodic serologic tests of the population should be performed [7]. It should be noted that interim results from a phase 3 trial of the Pfizer-BioNTech SARS-CoV-2 vaccine indicated 95% efficacy in preventing COVID-19 [10]. However, a rapid decline in both immunogenicity [11] and vaccine effectiveness [12] suggests that periodic serological tests will still be required during the post-pandemic period.

To detect SARS-CoV-2 antibodies in serum or whole blood, lateral flow immunoassays (LIFA) or paper-based assays are commonly used [1]. These tests are rapid (~10–20 min) and easy to use, but their sensitivity is low. Chemiluminescence immunoassays (CLIA) tests are more sensitive and have higher throughput [13,14], but they require bulky and expensive systems that are typically not available in limited resource settings. A more cost-effective test, the enzyme-linked immunosorbent assay (ELISA) has higher sensitivity than the lateral flow assays and allows processing of multiple samples simultaneously. However, ELISA is very time consuming (~245 min) [15] and laborious [16]. Overall, new technologies are needed to rapidly screen multiple samples for anti-SARS-CoV-2 antibodies with highly sensitivity, economy, and operational simplicity.

**Figure 1:**
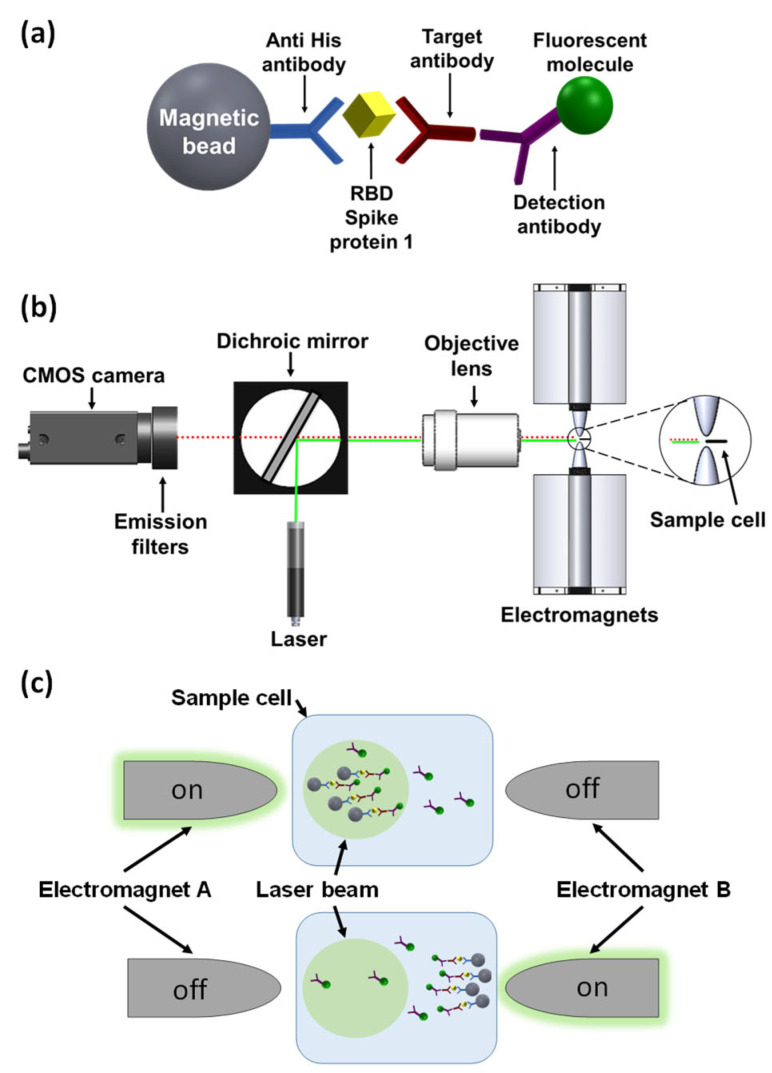
Magnetic modulation biosensing (MMB) system and SARS-CoV-2 serological assay illustration. (**a**) The SARS-CoV-2 serological assay includes a magnetic bead coated with anti-His antibodies that attach the receptor-binding domain (RBD) of the SARS-CoV-2 spike protein 1 (S1)-His tag, a target anti-SARS-CoV-2 S1 IgG antibody, and a fluorescently labeled detection antibody. (**b**) Schematic of the MMB system. A 532 nm laser beam is reshaped using two plano-convex lenses (not shown), reflected by a dichroic mirror, and focused by an objective lens onto a borosilicate glass cuvette positioned between two electromagnets. The emitted flashing fluorescence is collected by the same objective lens, filtered by two emission filters, and detected by a CMOS camera. (**c**) Using an alternating magnetic field gradient, two electromagnets (A and B), located on each side of the sample cell, aggregate and move the magnetic beads with target antibodies and fluorescently labeled detection antibodies from side to side, in and out of the laser beam. When electromagnet A is on, the clump of beads enters the laser beam, and the fluorescence emission is detected. When electromagnet B is on, the clump of beads exits the laser beam and background emission is detected.

Previously, we introduced a novel platform, termed magnetic modulation biosensing (MMB), that can rapidly detect low concentrations of fluorescently labeled target molecules, such as proteins and antibodies in blood [17,18,19,20,21]. Here, using the MMB platform, we present a rapid, highly sensitive, and specific anti-SARS-CoV-2 IgG serological assay. SARS-CoV-2 has four structural proteins, namely the spike (S), membrane (M), envelope (E), and nucleocapsid (N) proteins [22,23]. The spike (S) protein contains two subunits, S1 and S2, that mediate the entry of the virus into the host cells. The S1 subunit contains a receptor-binding domain (RBD) that recognizes the angiotensin-converting enzyme 2 (ACE2) receptor. The S2 subunit contains a hydrophobic fusion peptide and two heptad repeat regions responsible for membrane fusion [24]. Once the S1 binds to the host receptor, the S2 changes its conformation and facilitates fusion between the viral envelope and the host cell membrane, allowing the entry of viral genetic information. Therefore, the SARS-CoV-2 spike protein (S), specifically the RBD in the S1 subunit, is a crucial target for the immune system in inducing antibodies to protect against SARS-CoV-2 virus infection [25,26]. In an MMB-based anti-SARS-CoV-2 IgG serological assay, magnetic beads are attached to the RBD, which captures the anti-SARS-CoV-2 IgG antibodies. Then, a second, fluorescently labeled antibody is added to form a “sandwich” assay with the analyte and the capture protein (Figure 1a).

By blindly testing serum samples from RT-qPCR SARS-CoV-2-positive and healthy patients, we demonstrated the clinical sensitivity and specificity of the assay and compared it to the gold standard ELISA test. Most notably, compared with the ELISA test, the MMB-based assay detected an increase in the IgG concentration much earlier after vaccination. Moreover, the MMB-based assay had much higher sensitivity in detecting IgG levels in RT-qPCR SARS-CoV-2-positive samples that ELISA had identified as negative or borderline. This simple-to-use and highly sensitive method can improve serological tests and be used to screen populations that ELISA might falsely identify as negative (e.g., asymptomatic individuals or those very recently vaccinated).

## 2. Materials and Methods

### 2.1. Magnetic Modulation Biosensing (MMB) Principles and Assay Procedure

In an MMB-based SARS-CoV-2 serological assay (Figure 1a), magnetic beads (M-280; Thermo Fisher, Carlsbad, CA, USA) are conjugated to His tag monoclonal antibodies (10 µg, 70796-3, Novagen, Madison, WI, USA) according to the manufacturer’s protocol, and mixed overnight at 4 °C with the recombinant SARS-CoV-2 receptor-binding domain (RBD) that captures the target antibodies. Then, a second fluorescently labeled detection antibody is added to form a “sandwich” assay with the antibody and the RBD. To increase the sensitivity of fluorescence detection, two electromagnets (Figure 1b,c) generate alternating magnetic field gradients that condense the magnetic beads into a small volume and set them in periodic motion in and out of a laser beam. Every time the beads cross the laser beam, the fluorescently labeled detection antibodies generate a fluorescent signal that is detected by a camera. The amplitude of the periodic fluorescence signal is directly proportional to the number of the target molecules in the sample. Moreover, the aggregation of the magnetic beads from the entire sample volume into the detection area significantly increases the signal. Hence, MMB-based assays are characterized by both high sensitivity, quantitative results, and a short protocol [18,19,20,21].

### 2.2. MMB Optical Setup

In MMB (Figure 1b), a 3.5 mm diameter beam from a 532 nm laser diode module (CPS532, Thorlabs, Newton, NJ, USA), working at 0.25 mW, is focused to a 150 µm spot size by a pair of plano-convex lenses (ThorLabs, 200 and 50 mm focal lengths) and an objective lens (Newport, M-10X, 0.25 NA). The beam, diverted by a dichroic mirror (BrightLine Di02-R532, Semrock, Rochester, NY, USA) to the objective lens, exits the objective lens and is focused on a rectangular borosilicate sample cell (W2540, Vitrocom, Mountain Lakes, NJ, USA) containing the fluorescently tagged beads. The modulation frequency of the electromagnets is set to 1 Hz. The emitted fluorescence is collected using the same objective lens, passed through the dichroic mirror and two emission filters (FF03-575/25, Semrock, Rochester, NY, USA), and detected by a digital camera (GS3-U3-23S6M-C, FLIR, Wilsonville, OR, USA). A sequence of 600 images is acquired over a period of 12 s. The mean grey value (MGV) from the laser beam area of each image is calculated, and the peak-to-peak MGV differences over time are measured and averaged.

### 2.3. Sample Collection

The study in this research included 85 positive serum samples (Supplementary Material, Table S1a) from RT-qPCR SARS-CoV-2-positive patients and 79 negative serum samples (Supplementary Material, Table S1b) from patients presenting to Sheba Medical Center in 2019 (before the COVID-19 outbreak). To evaluate how soon the MMB-based assay can detect an increases in IgG concentrations following vaccination, 40 serum samples (Supplementary Material, Table S2) were taken from 10 individuals, who were in the process of being vaccinated with two doses of BNT162b2 mRNA (Pfizer-BioNTech) 21 days apart. The samples were collected at 7, 14, 21, and 28 days following the first dose. In addition, the research included 25 RT-qPCR SARS-CoV-2-positive samples (Supplementary Material, Table S3) that were taken from a large cohort study [15] and had been identified as borderline negative by the ELISA test.

All samples were obtained from the National Center for Zoonotic Viruses at the Central Virology Laboratory of the Ministry of Health at Sheba Medical Center, Israel. The SARS-CoV-2-positive samples were collected from Israeli patients presenting to Sheba Medical Center, Tel-HaShomer, and were diagnosed by RT-qPCR according to the World Health Organization (WHO) protocol.

Patient information, including age, gender, country of origin, country of acquisition, and the day the sample was taken after the first RT-qPCR-positive result, was obtained from the electronic medical record. All the samples (20 μL each) were stored at −20 °C, delivered to Bar-Ilan University on dry ice, and then thawed once for the MMB assay. The study was approved by the Institutional Review Board of Sheba Medical Center.

### 2.4. Serological Assays

#### 2.4.1. ELISA Immunoglobulin (Ig)G Anti- SARS-CoV-2 S1 Dose Response

To detect anti SARS-CoV-2 IgG antibodies, two 96-well plates (M9410, Nunc-Immuno, Thermo Fischer, Carlsbad, CA, USA) were pre-coated overnight at 4 °C with 100 µL per well of 1 µg/mL RBD of the SARS-CoV-2 spike protein 1. The plates were then washed and blocked for 60 min at room temperature with 3% DifcoTM skimmed milk (232100, BD Biosciences, Franklin Lakes, NJ, USA). Following the initial incubation, the blocking solution was removed, and the two plates were incubated for 120 and 30 min, respectively, at room temperature with increasing concentrations, ranging from 0–$10^{7}$ ng/L, of a recombinant human IgG anti-SARS-CoV-2 S1 monoclonal antibody (CR3022, Native Antigen) diluted in 1% skim milk. Then, the plates were washed (three times with washing buffer, PBS with 0.05% Tween20) and incubated at room temperature for 60 and 15 min, respectively, with 50 µL of goat anti-human IgG horseradish peroxidase (HRP) conjugate (115-035-071, Jackson ImmunoResearch) (diluted 1:15,000 with 1% skimmed milk). The plates were then washed again three times with the washing buffer, PBS with 0.05% Tween 20. To visualize the reactions, 100 μL of a chromogenic substrate (TMB, ab171523, Abcam, Cambridge, UK) were added to the plates for 5 min. Finally, the reactions were stopped by adding 100 μL of 1M hydrochloric acid. The optical density (OD) of each reaction was measured at 450 nm using an ELISA plate reader (Sunrise^TM^, Tecan, Männedorf, Switzerland). The total assay times for the two plates were 245 and 110 min, respectively (not including washing steps). The blank measurement was repeated six times (n=6), and the number of repetitions for all other concentrations was three (n=3).

To further analyze the analytical sensitivity of the 245-min ELISA test, we performed a dose response test using the WHO international standard for anti-SARS-CoV-2 S1 IgG antibody in concentrations of 0, 0.98, 1.95, 7.8, 15.6, 31.25, 62.5, 125, 250 and 500 IU/mL. The number of repetitions at each concentration was two (n=2).

#### 2.4.2. Magnetic Modulation Biosensing Immunoglobulin (Ig)G Anti- SARS-CoV-2 RBD Dose Response

To determine the analytical sensitivity, limit of detection (LoD), and dynamic range of the MMB-based SARS-CoV-2 IgG assay, we measured increasing concentrations of a recombinant human IgG anti-SARS-CoV-2 S1 monoclonal antibody (CR3022, Native Antigen, Kidlington, United Kingdom) in the MMB system. Using Thermo Fisher’s standard coupling procedures, we pre-conjugated tosylactivated magnetic beads (M-280; Thermo Fisher, Carlsbad, CA, USA) to an anti-His tag antibody (Novagen, Madison, WI, USA) and incubated them overnight with S1 RBD-His tag (obtained from the National Center for Zoonotic Viruses at the Central Virology Laboratory). The conjugated beads were incubated for 20, 30, 60, or 120 min at room temperature with increasing concentrations of human IgG anti-SARS-CoV-2 S1 monoclonal antibodies, ranging from 0–$10^{7}$ ng/L. The initial incubation was followed by incubation with a fluorescently labeled detection antibody (ab7005, Donkey F(ab′)2 Anti-Human IgG-H&L (PE), Abcam, Cambridge, United Kingdom), for 10, 15, 30, or 60 min. Thus, the total assay times, not including washing steps, were 30, 45, 90 and 180 min. To remove unbound detection antibodies, a single buffer replacement was performed after each incubation. The final solution was then loaded into a borosilicate glass cuvette and measured in the MMB system. Using the 30-, 90- and 180-min assays, the number of repetitions of the blank measurement was six (n=6); for all other concentrations, it was three (n=3). Using the 45-min assay, we performed a total of three experiments over the course of three days. Overall, the number of repetitions of the blank measurement was 13 (n=13). The numbers of repetitions for all other concentrations were between 8 and 13 (n=8–13). The reaction buffer solution was a mixture of phosphate-buffered saline (× 1), 1 mg/mL bovine serum albumin (BSA) (Sigma), and 0.05% Tween20 (Sigma-Aldrich). To provide a fair comparison between the ELISA test and the MMB-based assay, in both assays, we used the same RBD of the spike protein 1 of SARS-CoV-2 as a capture antigen and used the human IgG anti-SARS-CoV-2 S1 monoclonal antibody as a target.

To further analyze the analytical sensitivity of the 45-min MMB-based assay, we performed a dose response test using the WHO international standard for anti-SARS-CoV-2 S1 IgG antibody in concentrations of 0, 0.98, 1.95, 7.8, 15.6, 31.25, 62.5, 125, 250 and 500 IU/mL. The number of repetitions of the blank measurement was six (n=6); for all other concentrations, it was three (n=3).

### 2.5. Clinical Sensitivity and Specificity of the MMB-Based SARS-CoV-2 IgG Immunoassay

To determine the receiver operating characteristic (ROC) cutoff for the MMB-based SARS-CoV-2 IgG assay, we blindly tested 85 SARS-COV-2-positive serum samples (Supplementary Materials, Table S1a) that had been collected at least 14 days following a positive RT-qPCR test. We also blindly tested 79 SARS-CoV-2-negative serum samples (Supplementary Materials, Table S1b) retrieved from patients presenting to Sheba Medical Center in 2019 (i.e., before the COVID-19 outbreak). The MMB-based SARS-CoV-2 IgG assay used 60,000 beads that were incubated for 30 min at room temperature with 2 μL of serum sample diluted in 18 μL of buffer solution (PBS with 0.05% Tween20 and 1% BSA). After a single washing step, the fluorescently labeled detection antibody (Donkey F(ab’)2 Anti-Human IgG - H&L (PE), Abcam) was added to the beads for 15 min, followed by another washing step. The final solution was measured using the MMB system, and the total assay time was 45 min. Statistical analyses were performed using GraphPad Prism 7 (GraphPad Software, Inc., San Diego, CA, USA).

To further evaluate the clinical performance of the 45-min MMB-based assay and see how soon after vaccination it can detect an increase in the antibody concentration, we measured the time courses of IgG levels for 10 individuals who were undergoing vaccination with two doses of BNT162b2 mRNA 21 days apart, and compared the results to those of the 245-min SARS-CoV-2 ELISA test. The samples were collected 7, 14, 21 and 28 days following the first dose. In addition, we measured 25 patients’ samples that were taken from a large cohort study [15]. These samples had tested positive for SARS-CoV-2 using RT-qPCR but were identified as negative or borderline using the ELISA test.

### 2.6. Data Analysis

The clinical sensitivities of the MMB and ELISA tests for SARS-CoV-2 were calculated as the percentages of SARS-CoV-2-positive patients that were identified as positive by each assay. This measurement of clinical sensitivity should be distinguished from the analytical sensitivity of a detection system, which is the ability of the system to produce a change in signal for a defined change in the quantity being measured. It is also different from the limit of detection (LoD), which is the minimum number of target molecules that can be detected by the detection system [27]. The LoD for each set of experiments was calculated as three standard deviations over the blank measurement. Specificity was calculated as the percentage of healthy patients that were identified as negative by the assay. Statistical analyses were performed using GraphPad Prism 7 (GraphPad Software, Inc.). The dynamic range was calculated as the range in which the response changed when the analyte concentration was changed. The linear range was calculated as the range of concentrations in which the signals were directly proportional to the concentration of the analyte in the sample.

## 3. Results

### 3.1. Analytical Performance of the MMB-Based SARS-CoV-2 IgG Assay 

**Figure 2:**
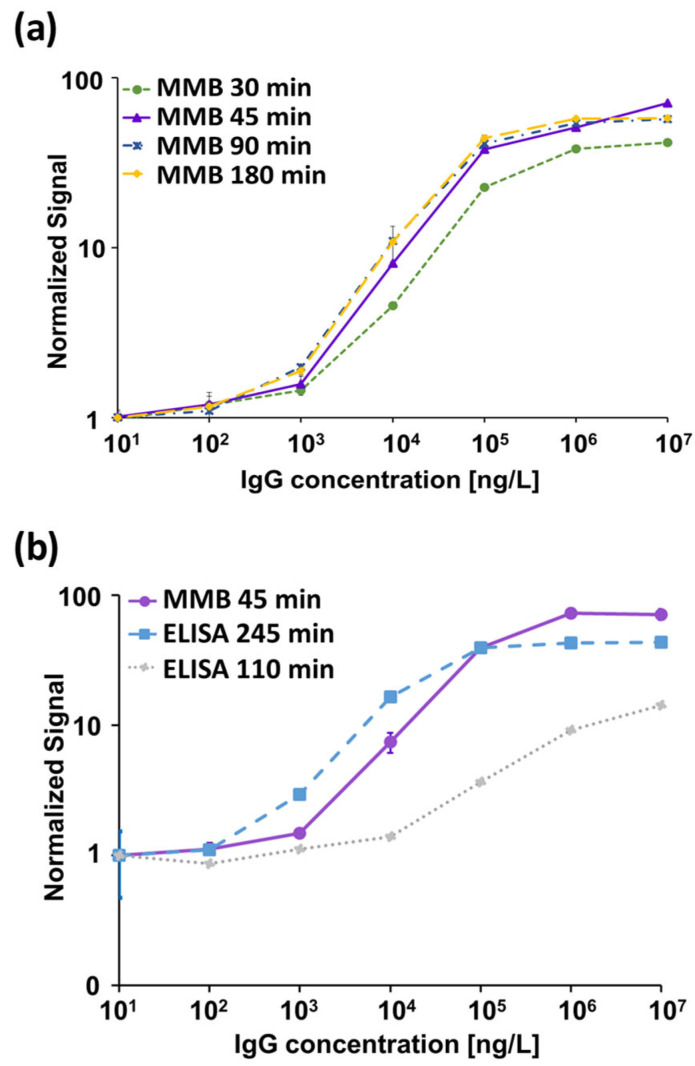
Dose response of a recombinant human immunoglobulin (Ig)G anti-SARS-CoV-2 S1 monoclonal antibody. (**a**) MMB-based dose response for 30-, 45-, 90- and 180-min assay times. The calculated limits of detection (LoD) are 593, 129, 187 and 144 ng/L, respectively. (**b**) Dose response comparison between the 45-min MMB-based assay and the 110- and 245-min ELISA tests. The calculated LoDs of the ELISA tests are 6267 and 817 ng/L, respectively. Error bars represent the standard deviations of 6–13 measurements at the blank concentration (n=6–13), and 3–13 measurements at all other concentrations (n=3–13).

A serologic assay’s sensitivity and specificity depend on the specificity of the capture antigen and the sensitivity of the detection system. Here, we used RBD as a capture antigen and a human IgG anti-SARS-CoV-2 S1 monoclonal antibody as our target. To obtain the optimal analytical performance of the MMB-based assay, we examined different assay incubation times (Figure 2a) and calculated the resulting LoD for each experiment. The dose-response curves of the 30-, 45-, 90- and 180-min assays showed LoDs of 593, 129, 187 and 144 ng/L, respectively. All MMB-based assays had a 4-log dynamic range and a 2-log linear range. The analytical sensitivity over the assays’ linear range was ~21–43 × 10^−5^ (normalized fluorescence signal/[ng/mL]). Based on these results, the optimal duration of the MMB-based assay was selected to be 45 min.

In addition, we compared the analytical performance of the 45-min MMB-based assay with the 110- and 245-min ELISA tests (Figure 2b). The LoDs of the 110- and 245-min ELISA tests were 6267 and 817 ng/L, respectively. Both ELISA tests had a 3-log dynamic range and a 1-log linear range. The analytical sensitivities of the 110- and 245-min ELISA tests over their respective linear ranges were 2.5 × 10^−5^ and 37 × 10^−5^ (normalized absorbance signal/[ng/L]).

**Figure 3:**
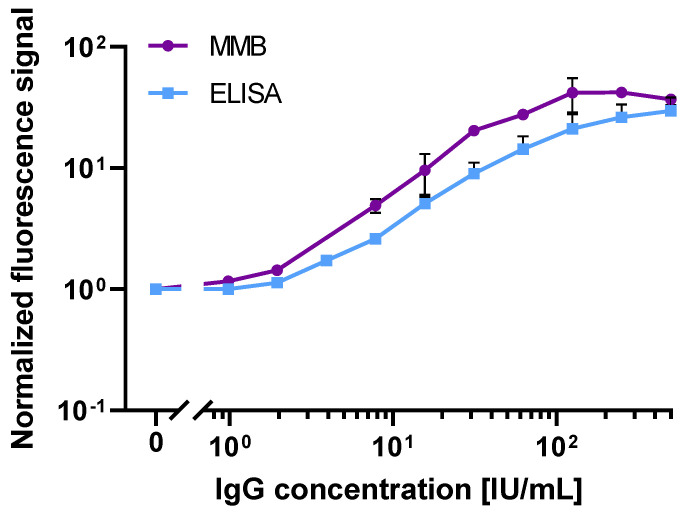
Dose response of WHO International Standard recombinant human immunoglobulin (Ig)G anti-SARS-CoV-2 S1 antibody. Dose response comparison between the 45-min MMB-based assay and the 245-min ELISA tests. The calculated LoDs of the MMB and the ELISA tests are 1.14 and 4.35 IU/mL, respectively. Error bars represent the standard deviations of 2–6 measurements at all concentrations (n=2–6). Abbreviations: IU, International Unites.

To further compare the analytical sensitivities of the 245-min ELISA test and 45-min MMB-based serological assay, we used the WHO international standard for anti-SARS-CoV-2 S1 IgG antibody (Figure 3). Both assays had a 2-log dynamic range. However, compared to the ELISA test, the MMB-based assay had an ~3.8-fold lower LoD (1.14 vs. 4.35 IU/mL) and an ~3-fold higher analytical sensitivity (0.65 vs. 0.21 normalized absorbance signal/[IU/mL]). Moreover, in the ELISA test, only values above 1.1 (ELISA absorbance signal) are regarded as positive, so it can positively detect the WHO international standard for anti-SARS-CoV-2 S1 IgG antibody at concentrations of 24.2 IU/mL. In comparison, the ROC cutoff of the MMB assay is 5.32 (MMB normalized signal), and thus the MMB assay can detect IgG concentrations of 8.4 IU/mL (an ~3-fold improvement over ELISA).

### 3.2. Clinical Sensitivity and Specificity of the Magnetic Modulation Biosensing SARS-CoV-2 Immunoassays 

**Figure 4:**
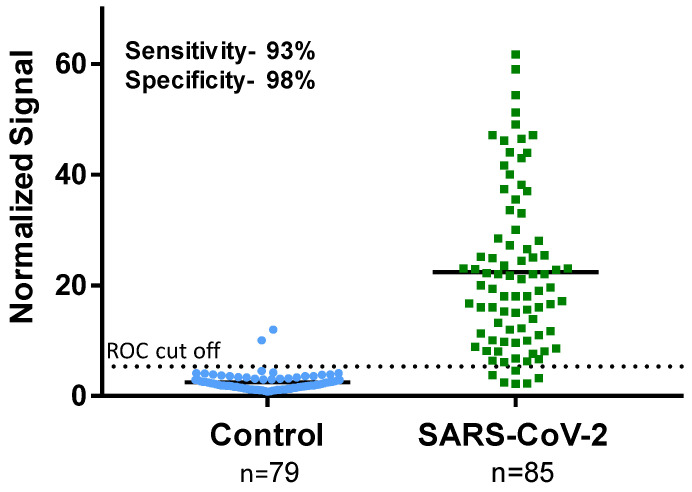
Sensitivity and specificity of the magnetic modulation biosensing SARS-CoV-2 immunoglobulin (Ig)G assay. The SARS-CoV-2-positive samples were confirmed as positive using RT-qPCR. The SARS-CoV-2-negative samples were retrieved from patients presenting to Sheba Medical Center in 2019 (before the COVID-19 outbreak). The receiver operating characteristic (ROC) cutoff for the MMB-based SARS-CoV-2 IgG assay is 5.32.

The 85 SARS-CoV-2-positive samples and 79 SARS-CoV-2-negative samples were blindly tested using the 45-min MMB-based SARS-CoV-2 IgG assay and 245-min IgG ELISA. Figure 4 shows the results of the 45-min MMB-based assay of clinical samples, which was able to detect 79 of 85 SARS-CoV-2-positive samples (93% sensitivity) and 77 of 79 SARS-CoV-2-negative samples (98% specificity). The 245-min IgG ELISA test was able to detect 78 of the 85 positive samples (92% sensitivity) and 78 of the 79 negative samples (99% specificity). It should be noted that one of the two samples that were falsely identified as positive by the MMB-based assay was also falsely identified as positive by the ELISA test (Supplementary Materials, Table S1).

### 3.3. Detecting Increases in IgG Concentrations Following Vaccination 

**Figure 5:**
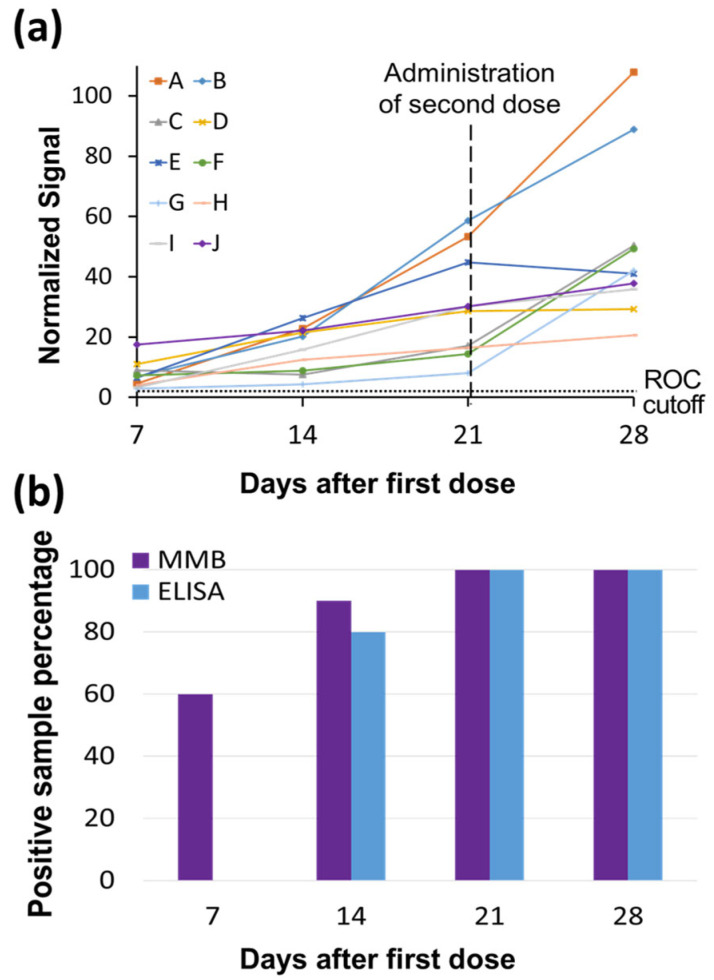
Time courses of SARS-CoV-2 immunoglobulin (Ig)G levels in vaccinated individuals. (**a**) Time courses of SARS-CoV-2 IgG levels of 10 individuals, labeled as A through J, who were vaccinated with two doses of BNT162b2 mRNA 21 days apart. The SARS-CoV-2 IgG levels were measured by the MMB-based assay. The blood samples were taken at 7, 14, 21 and 28 days after the first dose. The ROC cutoff for the MMB-based SARS-CoV-2 IgG assay is 5.32. (**b**) Percentage of SARS-CoV-2-positive IgG samples detected by the MMB and ELISA tests at the four different time points.

The time courses of the IgG levels of 10 individuals, vaccinated using two doses of BNT162b2 mRNA 21 days apart, are presented in Figure 4. In all cases, the IgG levels gradually increase over time (Figure 5a). Following the first shot, the 45-min MMB-based SARS-CoV-2 IgG assay identified as positive (i.e., having IgG levels above the ROC cutoff) 60% of the individuals on day 7, 90% of the individuals on day 14, and 100% of individuals on days 21 and 28. The 245-min SARS-CoV-2 ELISA test was able to detect IgG antibodies in 0%, 80%, 100%, and 100% of the individuals on days 7, 14, 21 and 28 following the first shot, respectively (Figure 5b, Supplementary Materials, Table S2). Seven days following the second shot (i.e., on day 28 following the first shot), both the 45-min MMB-based SARS-CoV-2 IgG assay and the 245-min SARS-CoV-2 ELISA test identified a significant increase in the antibody concentration in 50% of the individuals (5 out of 10).

### 3.4. Detecting Increases in IgG Concentrations Following Vaccination 

**Figure 6:**
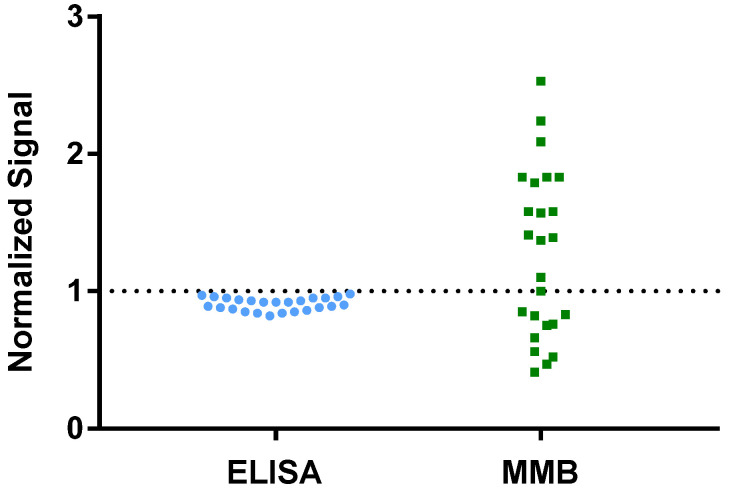
Analysis of 25 patients’ samples that had tested positive for SARS-CoV-2 using RT-qPCR but were identified as negative or borderline using the ELISA test. The raw signals of the ELISA- and MMB-based assay were normalized to the assays’ respective ROC cutoffs.

Table S3 (Supplementary Materials) lists 25 patients’ samples, taken from a large cohort study [15], that had tested positive for SARS-CoV-2 using RT-qPCR but were identified as negative or borderline using the ELISA test. The 45-min MMB-based SARS-CoV-2 IgG assay was able to detect 14 samples (56%) as positives (i.e., having IgG levels above the ROC cutoff). The signals of the ELISA- and MMB-based assays were normalized by the assays’ ROC cutoffs (Figure 6). The average normalized signal of the 14 samples that were identified as positive by the MMB-based assay was ~70% higher than the ROC cutoff.

## 4. Discussion

Serological assays are important for determining previous exposure to the SARS-CoV-2 virus in severely symptomatic, mildly symptomatic, and asymptomatic individuals. Accurately determining whether an individual has SARS-CoV-2 antibodies will help the authorities manage an efficient “immunity passport” policy, allowing individuals to travel internationally and safely return to work. In addition, screening to identify plasma donors who have a strong anti-SARS-CoV-2 antibody response (i.e., high-titer antibodies) can both help patients fight the disease and help develop antibody-based therapeutics and vaccines [3].

Compared with SARS-CoV-2 IgM/IgA antibodies, SARS-CoV-2 IgG antibodies remain detectable over at least several months and indicate long-term immunity [15,28,29]. Therefore, IgG antibodies provide information about virus epidemiology in the general population, and they can be detected in both symptomatic and asymptomatic individuals with previous COVID-19 infections. To demonstrate the clinical sensitivity of the MMB-based SARS-CoV-2 IgG assay, we carried out a clinical study and compared the results to the ELISA test, which is used by the Israel Central Virology Laboratory to measure the immunity level of present and past COVID-19-infected patients [15].

First, we determined the analytical performance of the MMB-based SARS-CoV-2 IgG assay by performing dose-response tests of a recombinant human IgG anti-S1 at four different incubation times: 30, 45, 90 and 180 min (Figure 2a). For the same target concentration (e.g., at $10^{5}$ ng/L), as the incubation time increases, a gradual increase in the fluorescent signal is observed. For the 90- and 180-min incubation times, the fluorescent signals remain approximately the same and the LoDs are similar (187 and 144 ng/L), suggesting that the assay reaches saturation following a 90-min incubation. In comparison, using the 30-min MMB-based assay, the fluorescent signal dropped by ~40% relative to the 90-min assay, and the LoD was only 593 ng/L. Compared with the 90- and 180-min assays, the 45-min MMB-based assay had a similar LoD (129 ng/L) and only an ~15% drop in signal. Thus, we concluded that the optimal incubation time is 45 min.

Second, we compared the analytical performance of the 45-min MMB-based SARS-CoV-2 IgG assay with the 245-min ELISA test (Figure 2b) using both a recombinant and the WHO international standard for anti-SARS-CoV-2 IgG antibodies. Using the recombinant anti-SARS-CoV-2 IgG antibody, the LoD of the ELISA test (817 ng/L) is ~6-fold higher than that of the MMB-based assay (129 ng/L), mainly due to the high standard deviation of the ELISA test’s measurements at low concentrations. Compared with the ELISA test, the MMB-based assay has much broader dynamic (4-log vs. 3-log) and linear ranges (2-log vs. 1-log), and similar analytical sensitivity (39 × 10^−5^ vs. 37 × 10^−5^ normalized signal/[ng/L]). Shortening the ELISA turnaround time to 110 min did not change the test’s dynamic and linear ranges, but it significantly reduced the analytical sensitivity (2.5 × 10^−5^ normalized signal/[ng/L]) and the LoD (6267 ng/L). The improved analytical performance of the MMB-based assay was also confirmed using the WHO international standard for the anti-SARS-CoV-2 IgG antibody. Compared with the 245-min ELISA test, the MMB-based assay has an ~3.8-fold lower LoD (1.14 IU/mL vs. 4.35 IU/mL), and an ~3-fold higher analytical sensitivity (0.65 vs. 0.21 normalized signal/[IU/mL]). Overall, the analytical performance of the 45-min MMB-based assay is better than the ELISA test, and is achieved with a ~5.4-fold improvement in the turnaround time (45 vs. 245 min). Using the WHO international standard for the anti-SARS-CoV-2 IgG antibody to compare the MMB-based assay and the ELISA test to other commercially available assays, such as Mindry, Roche, DiaSorin, Thermo-Fischer, and Euroimmun [30], the MMB-based assay has a similar dynamic range and a relatively low cutoff value (Supplementary Materials, Table S4).

Third, to determine the cutoff of the MMB-based assay, we compared the clinical performance of the 45-min MMB-based SARS-CoV-2 IgG assay with that of the 245-min ELISA test (Figure 4). Despite the shorter incubation time and faster overall turnaround time, the 45-min MMB-based assay achieved the same specificity (99% vs. 98%) and sensitivity (92% vs. 93%) as the gold standard 245-min ELISA test. Previously, using a large-scale clinical study, we compared the ELISA test to other commercially available serological assays (Roche, Abbott, Diasorin, BioMerieux, Beckman-Coulter, and Siemens) [31]. In that study, the ELISA test demonstrated the highest clinical sensitivity (89.4%) and lowest clinical specificity (97.8%) among the tested assays [31]. Here, the clinical sensitivity and specificity of the ELISA test (92% and 99%) were slightly higher than in the previous study (89.4% and 97.8%), which could be due to the small number of clinical samples.

Fourth, we showed that the MMB-based assay detects IgG antibodies significantly earlier after vaccination than ELISA (Figure 5). The ELISA test was able to detect IgG antibodies only from day 14 following the first vaccine dose onward, whereas the MMB-based assay detected IgG antibodies from as early as day 7. In particular, 6 of 10 serum samples that were taken 7 days following the first dose tested positive with the MMB-based assay but negative with the ELISA test. These results demonstrate that the MMB-based assay has a substantially broader detection window and higher sensitivity in vaccinated individuals also. In addition, the MMB-based SARS-CoV-2 IgG assay was able to correctly identify 14 samples (56%) out of 25 RT-qPCR SARS-CoV-2-positive samples from a large cohort study that were identified as negative or borderline using the ELISA test (Figure 6, Supplementary Materials, Table S3). Both the earlier detection of an increase in the antibody concentration and the identification of true-positives show that at low antibody concentrations, the MMB-based assay can better differentiate between SARS-CoV-2-positive and -negative patients. The improved analytical and clinical sensitivity of the MMB-based assay can help clinical laboratories provide critical information in a timely manner and help governments monitor the spread of the disease.

**Figure 7:**
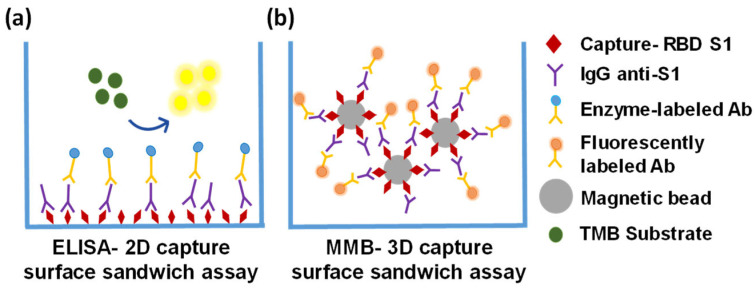
Schematic representations of the ELISA-2D and MMB-3D capture surfaces (**a**) The ELISA-2D capture surface is coated with a capture antigen (RBD of the SARS-CoV-2 spike protein 1 [RBD-S1]) that binds to any anti-SARS-CoV-2 IgG/IgM/IgA antibody in the sample. The antigen–antibody interactions are limited to a small volume above the surface of the plate. (**b**) The MMB-3D capture surface consists of magnetic beads conjugated to the capture antigen. The beads are free floating in the sample, providing more opportunities for antigen–antibody interactions.

The differences in the analytical and clinical performances of the MMB-based assay and the ELISA test can be attributed to several factors, such as the sources of the detection antibodies (the same antibodies were used in both assays, but they were purchased from different manufacturers), the buffer or blocking solution, the total interaction area, or the mobility and diffusion of the capture antigens in the solution. In a typical ELISA well, the covered surface area is ~80 ${\mathrm{mm}}^{2}$ (e.g., Greiner bio-one, Germany), and the capture antigen is immobilized on a 2D solid surface (Figure 7a), so the antigen–antibody interactions are limited to a small volume above the surface. In the MMB-based assay, the capture antigen is conjugated to magnetic beads that are free floating in the solution (Figure 7b). Although the covered surface area of the magnetic beads in each reaction (~60,000 beads, ~2.8 µm diameter each) is only ~1.5 ${\mathrm{mm}}^{2}$ (i.e., ~50 times smaller than a typical ELISA well), the diffusion of the beads in the MMB-based assay provides more opportunities for antigen–antibody interactions, resulting in a much shorter turnaround time.

Compared to the ELISA test, the MMB-based assay is simpler and less laborious. For example, unlike the ELISA test, which requires seven washing steps, the MMB-based assay requires only two washing steps. Moreover, to avoid high background noise in the ELISA test, the enzyme–substrate reaction has to be terminated by a stop solution and immediately followed by optical reading. Delays in the optical reading of the reaction can cause data drift and false results [32]. In contrast, in the MMB-based assay, delaying the optical reading will not change either the fluorescence signal or the results much. Overall, the ELISA test is considered to be a semi-quantitative method, whereas the MMB-based assay is a quantitative method. Additionally, the MMB-based assay requires minimal infrastructure, which enables simple operation in resource-limited settings.

One limitation of this study is that it is restricted to COVID-19 patients from Israel. Further research should examine whether there are differences in the immune responses of individuals from different countries or ethnicities. Future research is also required to evaluate the sensitivity and specificity of the MMB-based SARS-CoV-2 IgG assay on COVID-19-positive samples that were collected from patients from other countries and ethnicities, and possible different SARS-CoV-2 strains. In addition, cross-reactivity between antibodies against different viruses within the Coronaviridae family and other viral families should be examined.

## 5. Conclusions

In conclusion, this study presents a rapid, highly sensitive, and specific anti-SARS-CoV-2 IgG serological assay based on the MMB technology. Compared with the gold standard ELISA test, the MMB-based assay demonstrates an ~3.8–6-fold better limit of detection. In clinical tests, the MMB-based assay showed similar sensitivity (93% vs. 92%) and specificity (98% vs. 99%) as the ELISA test but managed to correctly detect 14 positive samples (56%) in a group of 25 RT-qPCR SARS-CoV-2-positive samples taken from a large cohort study and had been falsely identified as negatives using the ELISA test. In addition, it detected an increase in IgG antibody concentrations in vaccinated individuals much earlier with a much faster turnaround time (45 vs. 245 min).

The improved analytical and clinical sensitivities, lower LoD, shorter turnaround time, and simpler protocol of the MMB-based SARS-CoV-2 serological assay can help clinical laboratories provide rapid screening, improve critical information in a timely manner, and monitor the spread of the disease. In particular, accurately determining whether an individual has anti-SARS-CoV-2 IgG antibodies can help governments to efficiently implement an “immunity passport” and rebuild international tourism and travel. Moreover, this technology can be applied to other serological assays and significantly improves the field of serological diagnosis.

## Supplementary Materials

The following are available online at https://www.mdpi.com/article/10.3390/bios12010007/s1, Table S1: Data and characteristics of 85 SARS-CoV-2-positive and 79 SARS-CoV-2-negative samples tested using the 45-min MMB-based SARS-CoV-2 IgG assay (MMB) and the 245-min SARS-CoV-2 IgG ELISA test (ELISA); Table S2: Data and characteristics of 10 Israeli patients vaccinated with two doses of BNT162b2 mRNA (Pfizer-BioNTech) 21 days apart; Table S3: Data and characteristics of 25 Israeli patient’s RT-qPCR SARS-CoV-2-positive samples that were taken from a large cohort study and were identified as borderline negatives by the 245-min ELISA test; Table S4: Characteristics of anti-SARS-CoV-2 serological assays using WHO International Standard recombinant human immunoglobulin (Ig)G anti-SARS-CoV-2 S1 antibody: Manufacturer, target antigen, method, dynamic range, and cutoff value in BAU/mL.
