# Supplementary material for: Highly Sensitive and Specific SARS-CoV-2 Serological Assay Using a Magnetic Modulation Biosensing System
Source: Biosensors (Basel). 2021 Dec 23;12(1):7. doi: 10.3390/bios12010007 (PMC8773989; doi:10.3390/bios12010007)
Supplement: Supplementary file 1 [file biosensors-12-00007-s001.zip › biosensors-1478792-supplementary.pdf]

biosensors

MDPI

Supporting Materials

Highly Sensitive and Specific SARS-CoV-2 Serological Assay Using a Magnetic Modulation Biosensing System

Shira Avivi-Mintz [1], Yaniv Lustig [2,4], Victoria Indenbaum [2], Eli Schwartz [3,4] and Amos Danielli [1,*]

1 Faculty of Engineering, The Institute of Nanotechnology and Advanced Materials, Bar-Ilan University, Max and Anna Webb Street, 5290002, Ramat Gan, Israel; Shiravivi@gmail.com
2 Central Virology Laboratory, Israel Ministry of Health, Chaim Sheba Medical Centre, Tel-HaShomer, 5262000, Ramat Gan, Israel; yaniv.lustig@sheba.health.gov.il (Y.L.); Viki.Indenbaum@sheba.health.gov.il (V.I.)
3 The Center for Geographic Medicine, the Chaim Sheba Medical Center, Tel-Hashomer, 5262000, Ramat Gan, Israel; elischwa@tauex.tau.ac.il
4 Sackler faculty of Medicine, Tel Aviv University, 6997801, Tel Aviv, Israel
* Correspondence: amos.danielli@biu.ac.il

Table S1. Data and characteristics of 85 SARS-CoV-2-positive and 79 SARS-CoV-2-negative samples tested using the 45-minute MMB-based SARS-CoV-2 IgG assay (MMB) and the 245-minute SARS-CoV-2 IgG ELISA test (ELISA). a. Data analysis and characteristics of 85 RT-qPCR SARS-CoV-2-positive serum samples taken from Israeli patients presenting to Sheba Medical Center, Israel. b. Data analysis and characteristics of 79 SARS-CoV-2-negative samples taken from Israeli patients before the COVID-19 outbreak (before September 2019).

a).

Sample #	ELISA	MMB	Days from PCR	Sample #	ELISA	MMB	Days from PCR
**CO-854**	Pos (2.11)	Pos (6.38)	31	**CO-3869**	Pos (1.45)	Pos (8.61)	107
**CO-622**	Pos (5.61)	Pos (43.91)	21	**CO-3864**	Pos (2.84)	Pos (33.59)	122
**CO-623**	Pos (3.33)	Pos (15.23)	21	**CO-3813**	Pos (2.66)	Pos (11.31)	127
**CO-624**	Pos (3.3)	Pos (8.12)	10	**CO-3937**	Pos (4.42)	Pos (22.76)	132
**CO-625**	Pos (2.56)	Pos (24.89)	15	**CO-3936**	Pos (3.57)	Pos (15.62)	113
**CO-626**	Neg (0.2)	Neg (3.25)	16	**CO-3935**	Neg (0.13)	Pos (54.35)	134
**CO-636**	Neg (0.55)	Neg (4.56)	N.A.	**CO-3934**	Pos (5.55)	Pos (41.63)	130
**CO-627**	Pos (1.21)	Pos (12.22)	8	**CO-3933**	Pos (2.47)	Pos (13.24)	103
**CO-637**	Pos (8.5)	Pos (25.12)	24	**CO-3932**	Pos (5.79)	Pos (46.14)	123
**CO-843**	Pos (1.62)	Pos (11.67)	18	**CO-3931**	Pos (3.63)	Pos (13.91)	141
**CO-844**	Pos (7.85)	Pos (25.42)	34	**CO-3930**	Pos (6.02)	Pos (21.71)	117
**CO-846**	Pos (11.54)	Pos (61.67)	25	**CO-3929**	Pos (4.77)	Pos (35.47)	123
**CO-848**	Pos (2)	Pos (7.59)	20	**CO-3928**	Pos (2.73)	Pos (22.2)	124
**CO-849**	Pos (11.51)	Pos (47.12)	35	**CO-3927**	Pos (7.37)	Pos (51.2)	108
**CO-850**	Pos (1.23)	Pos (6.08)	38	**CO-3926**	Pos (2.78)	Pos (19.57)	107
**CO-851**	Neg (0.49)	Pos (6.26)	27	**CO-4302**	Pos (4.25)	Pos (27.21)	129
**CO-852**	Pos (3.71)	Pos (21.07)	28	**CO-7413**	Pos (2.06)	Pos (25)	75
**CO-853**	Neg (0.44)	Neg (2.4)	27	**CO-7419**	Pos (2.72)	Pos (22)	N.A.

**CO-3803**	Pos (1.18)	Pos (10.09)	112	**CO-7416**	Pos (4.37)	Pos (44)	N.A.
**CO-3801**	Pos (3.65)	Pos (19)	114	**CO-7396**	Pos (4.04)	Pos (37)	N.A.
**CO-3469**	Pos (1.64)	Pos (8.02)	119	**CO-7395**	Pos (2.97)	Pos (16)	N.A.
**CO-3372**	Pos (4.59)	Pos (11)	104	**CO-7389**	Pos (2.21)	Pos (18)	N.A.
**CO-3863**	Pos (2.31)	Pos (22.88)	127	**CO-7388**	Pos (3.21)	Pos (23)	N.A.
**CO-3862**	Pos (5.3)	Pos (28.46)	139	**CO-7387**	Pos (6.12)	Pos (22)	N.A.
**CO-3860**	Pos (6.23)	Pos (24.39)	117	**CO-7386**	Pos (4.99)	Pos (40)	N.A.
**CO-3858**	Pos (1.32)	Pos (6.8)	125	**CO-7412**	Pos (7.53)	Pos (59)	79
**CO-3857**	Pos (6.81)	Pos (38.13)	128	**CO-7411**	Pos (5.99)	Pos (28)	150
**CO-3856**	Pos (5.82)	Pos (16.74)	119	**CO-7410**	Pos (4.58)	Pos (49)	259
**CO-3854**	Neg (0.19)	Neg (3.77)	117	**CO-7408**	Pos (2.13)	Pos (18)	275
**CO-3851**	Neg (0.68)	Neg (2.23)	90	**CO-7407**	Pos (4.26)	Pos (8.1)	101
**CO-3812**	Pos (2.31)	Pos (6.67)	121	**CO-7404**	Pos (4.89)	Pos (33)	72
**CO-3811**	Pos (4.11)	Pos (19.35)	115	**CO-7402**	Pos (5.09)	Pos (30)	N.A.
**CO-3810**	Pos (3.96)	Pos (17.13)	121	**CO-7401**	Pos (4.25)	Pos (23)	N.A.
**CO-3808**	Pos (6.52)	Pos (23.56)	99	**CO-7397**	Pos (2.47)	Pos (16)	N.A.
**CO-3940**	Pos (3.51)	Pos (16.61)	139	**CO-7326**	Pos (2.07)	Pos (16)	76
**CO-3938**	Pos (3.84)	Pos (12)	127	**CO-7327**	Pos (6.62)	Pos (18)	264
**CO-3925**	Pos (6.73)	Pos (46.44)	95	**CO-7328**	Pos (6.7)	Pos (10)	267
**CO-3924**	Pos (5.72)	Pos (37.35)	107	**CO-7329**	Pos (5.04)	Pos (20)	267
**CO-3923**	Pos (6.1)	Pos (42.99)	143	**CO-7330**	Pos (3)	Pos (22)	267
**CO-3922**	Pos (1.68)	Neg (2.16)	58	**CO-7175**	Pos (2.17)	Pos (9.79)	54
**CO-3920**	Pos (5.62)	Pos (9.57)	121	**CO-6819**	Pos (2.81)	Pos (15)	120
**CO-3871**	Pos (1.51)	Pos (26.5)	90	**CO-7384**	Pos (2.73)	Pos (8.9)	N.A.
**CO-3919**	Pos (6.06)	Pos (47.1)	130				

b).

Sample #	ELISA	MMB	Sample #	ELISA	MMB
**5542/19**	Neg (0.31)	Neg (0.80)	**1397/19**	Neg (0.37)	Neg (3.38)
**5543/19**	Neg (0.44)	Neg (0.89)	**1438/19**	Equ (1.09)	Neg (3.57)
**5544/19**	Neg (0.32)	Neg (1.13)	**1263/19**	Neg (0.54)	Neg (2.53)
**5545/19**	Neg (0.31)	Neg (0.84)	**1266/19**	Neg (0.45)	Neg (3.37)
**5546/19**	Neg (0.31)	Neg (2.90)	**1278/19**	Neg (0.33)	Neg (3.56)
**5547/19**	Neg (0.38)	Neg (3.57)	**1301/19**	Neg (0.38)	Neg (1.34)
**5548/19**	Neg (0.32)	Neg (2.91)	**1312/19**	Neg (0.29)	Neg (3.86)
**5549/19**	Neg (0.37)	Neg (1.88)	**1334/19**	Neg (0.36)	Neg (3.68)
**5541/19**	Neg (0.37)	Neg (4.21)	**1343/19**	Neg (0.38)	Neg (2.04)
**5539/19**	Neg (0.33)	Neg (1.91)	**1346/19**	Neg (0.35)	Neg (3.15)
**5332/19**	Neg (0.24)	Neg (1.01)	**1391/19**	Neg (0.48)	Neg (3.13)
**5334/19**	Neg (0.2)	Neg (3.83)	**1158/19**	Neg (0.33)	Neg (2.68)
**5335/19**	Neg (0.31)	Neg (4.07)	**1053/19**	Neg (0.36)	Pos (12)

**5538/19**	Neg (0.32)	Neg (2.75)	**1112/19**	Neg (0.43)	Neg (2.4)
**5537/19**	Neg (0.32)	Neg (1.84)	**1189/19**	Neg (0.5)	Neg (2.25)
**5530/19**	Neg (0.24)	Neg (1.64)	**1242/19**	Neg (0.69)	Neg (1.79)
**5531/19**	Neg (0.43)	Neg (4.14)	**1206/19**	Neg (0.33)	Neg (1.5)
**5528/19**	Neg (0.3)	Neg (2.53)	**1262/19**	Neg (0.33)	Neg (1.2)
**5527/19**	Neg (0.32)	Neg (3.44)	**1063/19**	Neg (0.38)	Neg (2)
**5521/19**	Neg (0.35)	Neg (4.51)	**1119/19**	Pos (1.16)	Pos (10.08)
**5522/19**	Neg (0.5)	Neg (3.86)	**2193/19**	Neg (0.26)	Neg (1.11)
**5525/19**	Neg (0.27)	Neg (2.31)	**2217/19**	Neg (0.51)	Neg (1.36)
**5524/19**	Neg (0.46)	Neg (3.01)	**2327/19**	Neg (0.24)	Neg (1.5)
**5523/19**	Neg (0.4)	Neg (1.85)	**2156/19**	Neg (0.24)	Neg (1.11)
**5320/19**	Neg (0.49)	Neg (1.66)	**2278/19**	Neg (0.21)	Neg (1.21)
**5517/19**	Neg (0.46)	Neg (2.08)	**2321/19**	Neg (0.21)	Neg (1)
**5516/19**	Neg (0.28)	Neg (1.54)	**2325/19**	Neg (0.28)	Neg (0.95)
**5509/19**	Neg (0.36)	Neg (1.48)	**2328/19**	Neg (0.29)	Neg (1.17)
**5515/19**	Neg (0.32)	Neg (2.53)	**2246/19**	Neg (0.22)	Neg (1.11)
**5508/19**	Neg (0.43)	Neg (3.03)	**2244/19**	Neg (0.18)	Neg (1.10)
**5507/19**	Neg (0.74)	Neg (1.87)	**2252/19**	Neg (0.26)	Neg (2.5)
**5514/19**	Neg (0.27)	Neg (1.82)	**2521/19**	Neg (0.31)	Neg (2.2)
**5506/19**	Neg (0.73)	Neg (2.10)	**2324/19**	Neg (0.26)	Neg (4.1)
**5513/19**	Neg (0.46)	Neg (2.32)	**2323/19**	Neg (0.34)	Neg (1.3)
**5518/19**	Neg (0.36)	Neg (1.71)	**2211/19**	Neg (0.39)	Neg (2.66)
**5526/19**	Neg (0.37)	Neg (1.22)	**2251/19**	Neg (0.23)	Neg (1.9)
**5511/19**	Neg (0.51)	Neg (1.68)	**2158/19**	Neg (0.18)	Neg (1.8)
**5512/19**	Neg (0.53)	Neg (3.14)	**2260/19**	Neg (0.24)	Neg (1.6)
**5505/19**	Neg (0.54)	Neg (2.87)	**2326/19**	Neg (0.14)	Neg (2.4)
**5510/19**	Neg (0.39)	Neg (1.33)			

Using ELISA, values below 0.8 (ELISA absorbance units) are regarded as negative, values in the range of 0.8–1.1 are regarded as equivocal (which are clinically regarded as negative, but require further monitoring), and values above 1.1 are regarded as positive. Using the MMB-based assay, the ROC cutoff is 5.32. Thus, values below 5.32 (MMB normalized fluorescent signal) are regarded as negative, and values above 5.32 are regarded as positive. **Abbreviations:** ELISA, enzyme-linked immunosorbent assay; MMB, magnetic modulation biosensing; N.A., not applicable; Neg, negative; Pos, positive; Equ, Equivocal; ROC, receiver operating characteristic.

Table S2. Data and characteristics of 10 Israeli patients vaccinated with two doses of BNT162b2 mRNA (Pfizer-BioNTech) 21 days apart.

	Sample #	Age	Administration of first dose	Serology test (IgG)	ELISA	MMB
	**70001**			29/12/2020	Neg (0.24)	Neg (4.53)
**A**	**70302**	24	20/12/2020	04/01/2021	Pos (3.31)	Pos (22.76)
	**70720**			11/01/2021	Pos (4.32)	Pos (53.32)

	**71267**			19/01/2021	Pos (6.79)	Pos (107.83)
**B**	**70004**	38	20/12/2020	29/12/2020	Neg (0.41)	Pos (6.95)
	**70324**			04/01/2021	Pos (2.01)	Pos (20.18)
	**70715**			11/01/2021	Pos (1.89)	Pos (58.58)
	**71144**			18/01/2021	Pos (7.08)	Pos (88.83)
**C**	**70005**	66	20/12/2020	29/12/2020	Neg (0.27)	Pos (9.04)
	**70316**			04/01/2021	Equ (0.93)	Pos (7.5)
	**70706**			11/01/2021	Pos (1.41)	Pos (17.18)
	**71207**			19/01/2021	Pos (7.14)	Pos (50.41)
**D**	**70006**	53	20/12/2020	29/12/2020	Neg (0.38)	Pos (11.11)
	**70293**			04/01/2021	Pos (4.06)	Pos (21.52)
	**70670**			11/01/2021	Pos (3.54)	Pos (28.68)
	**71125**			18/01/2021	Pos (7.55)	Pos (29.26)
**E**	**70023**	44	20/12/2020	29/12/2020	Neg (0.29)	Pos (6.5)
	**70300**			04/01/2021	Pos (3.98)	Pos (26.3)
	**70688**			11/01/2021	Pos (5.1)	Pos (44.82)
	**71148**			18/01/2021	Pos (7.05)	Pos (41.02)
**F**	**70034**	61	20/12/2020	29/12/2020	Neg (0.24)	Pos (7.34)
	**70299**			04/01/2021	Pos (1.23)	Pos (8.84)
	**70683**			11/01/2021	Pos (1.58)	Pos (14.36)
	**71139**			18/01/2021	Pos (6.78)	Pos (49.35)
**G**	**70018**	67	20/12/2020	29/12/2020	Neg (0.29)	Neg (2.9)
	**70296**			04/01/2021	Neg (0.48)	Neg (4.35)
	**70679**			11/01/2021	Pos (1.23)	Pos (8.07)
	**72284**			N/A	Pos (7.38)	Pos (42.05)
**H**	**70044**	55	22/12/2020	29/12/2020	Neg (0.38)	Neg (4.07)
	**70493**			06/01/2021	Pos (2.33)	Pos (12.44)
	**70895**			13/01/2021	Pos (4.13)	Pos (16.53)
	**71383**			21/01/2021	Pos (6.08)	Pos (20.68)
**I**	**70048**	58	20/12/2020	29/12/2020	Neg (0.23)	Neg (3.32)
	**70310**			04/01/2021	Pos (2.03)	Pos (15.84)
	**70701**			11/01/2021	Pos (4.1)	Pos (32.2)
	**71170**			18/01/2021	Pos (7.12)	Pos (35.83)
**J**	**70075**	56	21/12/2020	30/12/2020	Neg (0.22)	Pos (17.56)
	**70375**			05/01/2021	Pos (3.71)	Pos (22.13)
	**70887**			13/01/2021	Pos (4.78)	Pos (30.15)
	**71461**			21/01/2021	Pos (7.66)	Pos (37.82)

The samples were obtained 7, 14, 21, and 28 days following the administration of the first dose. All patients received their second dose 21 days following the first dose. Using ELISA, values below 0.8 (ELISA absorbance units) are regarded as negative, values in the range of 0.8–1.1 are regarded as equivocal (which are clinically regarded as negative but

require further monitoring), and values above 1.1 are regarded as positive. Using the MMB-based assay, the ROC cutoff is 5.32. Thus, values below 5.32 (MMB normalized fluorescent signal) are regarded as negative and values above 5.32 are regarded as positive. Abbreviations: Neg, negative; Pos, positive; Equ, equivocal.

Table S3. Data and characteristics of 25 Israeli patient's RT-qPCR SARS-CoV-2-positive samples that were taken from a large cohort study and were identified as borderline negatives by the 245-minute ELISA test.

Sample #	ELISA	MMB	Days from PCR
**CO-926**	Equ (1.01)	Pos (7.3)	42
**CO-1486**	Equ (1.02)	Pos (9.74)	44
**CO-1472**	Equ (0.98)	Pos (9.73)	42
**CO-1488**	Equ (1.05)	Pos (13.48)	42
**CO-2301**	Equ (0.94)	Neg (3)	72
**CO-3362**	Equ (0.98)	Neg (4.07)	72
**CO-1436**	Equ (0.97)	Pos (9.5)	38
**CO-3368**	Equ (1.01)	Pos (9.71)	104
**CO-3573**	Equ (0.92)	Pos (8.35)	109
**CO-3673**	Equ (0.92)	Neg (4.53)	123
**CO-3675**	Equ (0.94)	Neg (4.36)	112
**CO-7409**	Equ (0.99)	Pos (11.9)	149
**CO-7128**	Equ (0.9)	Pos (11.1)	N.A.
**CO-6739**	Equ (1.07)	Neg (2.79)	N.A.
**CO-6718**	Equ (1.05)	Pos (8.4)	73
**CO-6722**	Equ (0.97)	Pos (5.83)	66
**CO-7147**	Equ (0.96)	Neg (3.52)	78
**CO-6655**	Equ (1.02)	Neg (4.43)	N.A.
**CO-4971**	Equ (0.95)	Neg (4)	N.A.
**CO-6757**	Equ (1.06)	Pos (8.4)	62
**CO-6759**	Equ (1.06)	Pos (7.4)	132
**CO-7063**	Equ (1.08)	Equ (5.32)	159
**CO-6635**	Equ (1.01)	Pos (7.48)	N.A.
**CO-7087**	Equ (1.03)	Neg (2.52)	236
**CO-7105**	Equ (1.04)	Neg (2.16)	N.A.
**CO-926**	Equ (1.01)	Pos (7.3)	42
**CO-1486**	Equ (1.02)	Pos (9.74)	44
**CO-1472**	Equ (0.98)	Pos (9.73)	42
**CO-1488**	Equ (1.05)	Pos (13.48)	42

The samples were tested using the 45-minute MMB-based SARS-CoV-2 IgG assay (MMB). Using ELISA, values below 0.8 (ELISA absorbance units) are regarded as negative, values in the range of 0.8–1.1 are regarded as equivocal (which are clinically regarded as negative but require further monitoring), and values above 1.1 are regarded as positive. Using the MMB-based assay, the ROC cutoff is 5.32. Thus, values below 5.32 (MMB normalized fluorescent signal) are regarded as negative and values above 5.32 are regarded as positive. **Abbreviations:** N.A., not applicable; Neg, negative; Pos, positive; Equ, equivocal.

Table S4. Characteristics of anti-SARS-CoV-2 serological assays using WHO International Standard recombinant human immunoglobulin (Ig)G anti-SARS CoV-2 S1 antibody: Manufacturer, target antigen, method, dynamic range, and cutoff value in BAU/mL.

Manufacturer name	Target antigen	Method	Dynamic range (BAU/mL)	Cutoff (BAU/mL)
MMB	S-RBD	MMB	1.14–125	8.4
RBD-ELISA	S-RBD	ELISA	4.35–250	24.2
Euroimmun	S1	ELISA	3.2–384	25.6 (25.6–35.2 borderline)
Diasorin	Trimeric S	CLIA	4.8–2080	33.8
Thermo Fisher	S1	FEIA	2.8–816	28 (28–40 borderline)
Roche	S-RBD	ECLIA	0.40–243	0.823
Snibe	S-RBD	CLIA	0.78–433	4.33
Mindary	S-RBD	CLIA	3.65–1216	12.16

BAU, binding antibody unit; CLIA, chemiluminescence immunoassay; ECLIA, electrochemiluminescence immunoassay; ELISA, enzyme-linked immunosorbent assay; FEIA, fluorescence enzyme immunoassay
